# Defect-Induced Luminescence Quenching vs. Charge Carrier Generation of Phosphorus Incorporated in Silicon Nanocrystals as Function of Size

**DOI:** 10.1038/s41598-017-01001-1

**Published:** 2017-04-13

**Authors:** Daniel Hiller, Julian López-Vidrier, Sebastian Gutsch, Margit Zacharias, Keita Nomoto, Dirk König

**Affiliations:** 1 Laboratory for Nanotechnology, Dept. of Microsystems Engineering (IMTEK), University of Freiburg, Freiburg, Germany; 2 School of Photovoltaic and Renewable Energy Engineering (SPREE), University of New South Wales (UNSW), Sydney, Australia; 3 Australian Centre for Microscopy and Microanalysis, The University of Sydney, Sydney, Australia; 4 Integrated Materials Design Centre (IMDC), University of New South Wales (UNSW), Sydney, Australia

## Abstract

Phosphorus doping of silicon nanostructures is a non-trivial task due to problems with confinement, self-purification and statistics of small numbers. Although P-atoms incorporated in Si nanostructures influence their optical and electrical properties, the existence of free majority carriers, as required to control electronic properties, is controversial. Here, we correlate structural, optical and electrical results of size-controlled, P-incorporating Si nanocrystals with simulation data to address the role of interstitial and substitutional P-atoms. Whereas atom probe tomography proves that P-incorporation scales with nanocrystal size, luminescence spectra indicate that even nanocrystals with several P-atoms still emit light. Current-voltage measurements demonstrate that majority carriers must be generated by field emission to overcome the P-ionization energies of 110–260 meV. In absence of electrical fields at room temperature, no significant free carrier densities are present, which disproves the concept of luminescence quenching via Auger recombination. Instead, we propose non-radiative recombination via interstitial-P induced states as quenching mechanism. Since only substitutional-P provides occupied states near the Si conduction band, we use the electrically measured carrier density to derive formation energies of ~400 meV for P-atoms on Si nanocrystal lattice sites. Based on these results we conclude that ultrasmall Si nanovolumes cannot be efficiently P-doped.

## Introduction

Since the first reports about (heavy) P-doping of silicon nanocrystals (Si NCs), luminescence quenching was often attributed to non-radiative exciton recombination with P-induced free carriers (Auger recombination)^1^. Only few works addressed alternative quenching mechanisms like defects^2–4^. We note that a direct proof of successful P-doping is nontrivial since a true free carrier is not generated due to its confinement in the quantum dot (QD). One important parameter in the investigation of P-doped, oxide-embedded Si NCs is the excess Si concentration that determines the size and separation of NCs as well as the degree of agglomeration. While isolated and mainly spherical NCs are formed at low Si concentrations, excess Si contents above the percolation threshold form highly irregular agglomerated Si NC networks. The threshold that separates these regimes is SiO_x≈0.6_ for very thin films in a superlattice (SL) and SiO_x≲1_ for thick bulk films^5–7^. While the investigation of P-doping of small and well-separated Si NCs remains a challenging task, it does not come as a surprise that extended Si NC networks can be doped successfully^8, 9^. Another important parameter is the dopant concentration and the term doping itself. The latter requires a disambiguation for crystallites at the bottom end of the nanoscale where it is often used deceptively for: (i) the bare incorporation of P-atoms into nanocrystals, (ii) the observation of optical or electrical effects caused by P-incorporation, and (iii) the actual generation of free majority charge carriers by ionization of the dopant at room temperature. We refer to the latter process as *electronic doping*. Thereby, we discriminate the delivery of free charge carriers from other processes induced directly or indirectly by the presence of foreign atoms within or adjacent to nanocrystals. Electronic doping of bulk semiconductors involves typically impurity concentrations in the ppm-range or less, which inevitably increases to the sub-percent range in nanocrystals consisting of just hundreds or thousands of atoms. However, doping levels of 10 at% and more^10–12^ are not relevant for electronic doping, though there is justified interest in the induced plasmonic properties^11, 12^. In general, such impurity levels constitute alloying rather than doping and are sometimes termed hyper-doping. At typical Si NC processing temperatures the bulk Si solubility limit for P is slightly below 1 at%,^13^ while the bulk semiconductor-metal transition is observed in the at‰ range^14, 15^. Due to self-organized growth of all P-doped Si NCs, there is no homogeneous impurity concentration of e.g. 1 P-atom in every NC. P-doping of Si NCs is governed by a distribution. Moreover, the integer number of incorporated P-atoms makes the doping concentration quantized and not an arbitrarily selectable fabrication parameter. There is consensus that P-atoms can be incorporated even in small Si NCs, irrespective of fabrication method^16–19^ and self-purification mechanisms^20^. However, the exact position of these incorporated P-atoms in the NC (interstitial vs. substitutional, near-surface vs. core) and their impact on optical and electrical properties of the Si NC are still under debate. A comprehensive review about this topic is given by ref. 21.

In this study, we correlate the structural, optical and electrical properties of size-controlled and well-separated P-incorporating Si NCs. Atom probe tomography (APT) is used to determine the distribution of P-atoms as function of NC size within each sample and the size distribution of the P-incorporating and P-free fraction of the NC ensemble. Combined photoluminescence (PL) and current-voltage (I-V) measurements, supported by density functional theory (DFT) results, reveal the formation energies of substitutional P and the ionization energies required to generate majority charge carriers.

## Results

### Structural analysis of P-incorporation as function of NC size

For this study, 2 sample sets (labelled A and E) of SiO_2_-embedded size-controlled Si NCs with mean sizes from 1.9 to 3.7 nm were prepared using P-doped Si-rich oxynitride (SRON)/SiO_2_ superlattices deposited by plasma-enhanced chemical vapour deposition (PECVD). Highly Ar-diluted PH_3_ was used to deposit SRON:P layers with nominal P-concentrations C_P_ of 0.18–0.71 at%. For sample set A, dedicated to APT measurements, the P-concentration of SRON:P was kept constant at the highest level of 0.71 at%. The P-concentration of sample set E (consisting of P-doped samples labelled P and intrinsic reference samples labelled R) is dedicated to electrical measurements such as I-V and I-t and was selected for each SRON:P thickness so that on average approximately 1 P-atom is incorporated in each NC. For this selection, we used APT data of sample set A (cf. ref. 17). Since samples with larger NCs tend to incorporate more P-atoms, we reduced the PH_3_ flux for samples with thicker SRON:P layers, making use of the linear relationship between PH_3_ flux and P-concentration (cf. Supplementary Fig. S1 and Table 1).

**Table 1:** Data values of the structural and electrical characterization.

Sample	t_SRON(:P)_ (nm)	C_P_ at%	d_NC_ (nm)	ρ_NC_ (10^12^ cm^−2^)	[P]_APT_	E_peak_ (MV/cm)
A2	2	0.71	1.9	0.8 ± 0.1	1.01 ± 1.6	
A3	3	0.71	2.4	1.1 ± 0.2	1.47 ± 2.2	
A4	4	0.71	3.0	1.5 ± 0.2	2.97 ± 3.5	
A5	5	0.71	3.7	1.6 ± 0.2	5.43 ± 5.8	
P2	2	0.71	*1.9*	*0.8* ± *0.1*	*1.01* ± *1.6*	~0.6
P3	3	0.48	*2.4*	*1.1* ± *0.2*	*0.99* ± *1.5*	0.315
P4	4	0.18	*3.0*	*1.5* ± *0.2*	*0.75* ± *0.9*	0.150
P5	5	0.18	*3.7*	*1.6* ± *0.2*	*1.37* ± *1.5*	0.115
R2	2	0	*1.9*	*0.8* ± *0.1*	0	–
R3	3	0	*2.4*	*1.1* ± *0.2*	0	–
R4	4	0	*3.0*	*1.5* ± *0.2*	0	–
R5	5	0	*3.7*	*1.6* ± *0.2*	0	–

Here, t_SRON(:P)_ denotes the Si-rich oxide layer thickness, C_P_ the nominal P-concentration in SRON:P, d_NC_ the mean NC size, ρ_NC_ the areal density of NCs per cm^²^ and SL layer, [P]_APT_ the average number of P-atoms per NC, and E_peak_ the electric field of the J-peak in Fig. 3a. Extrapolated values are in *italic*.

**Figure 1:**
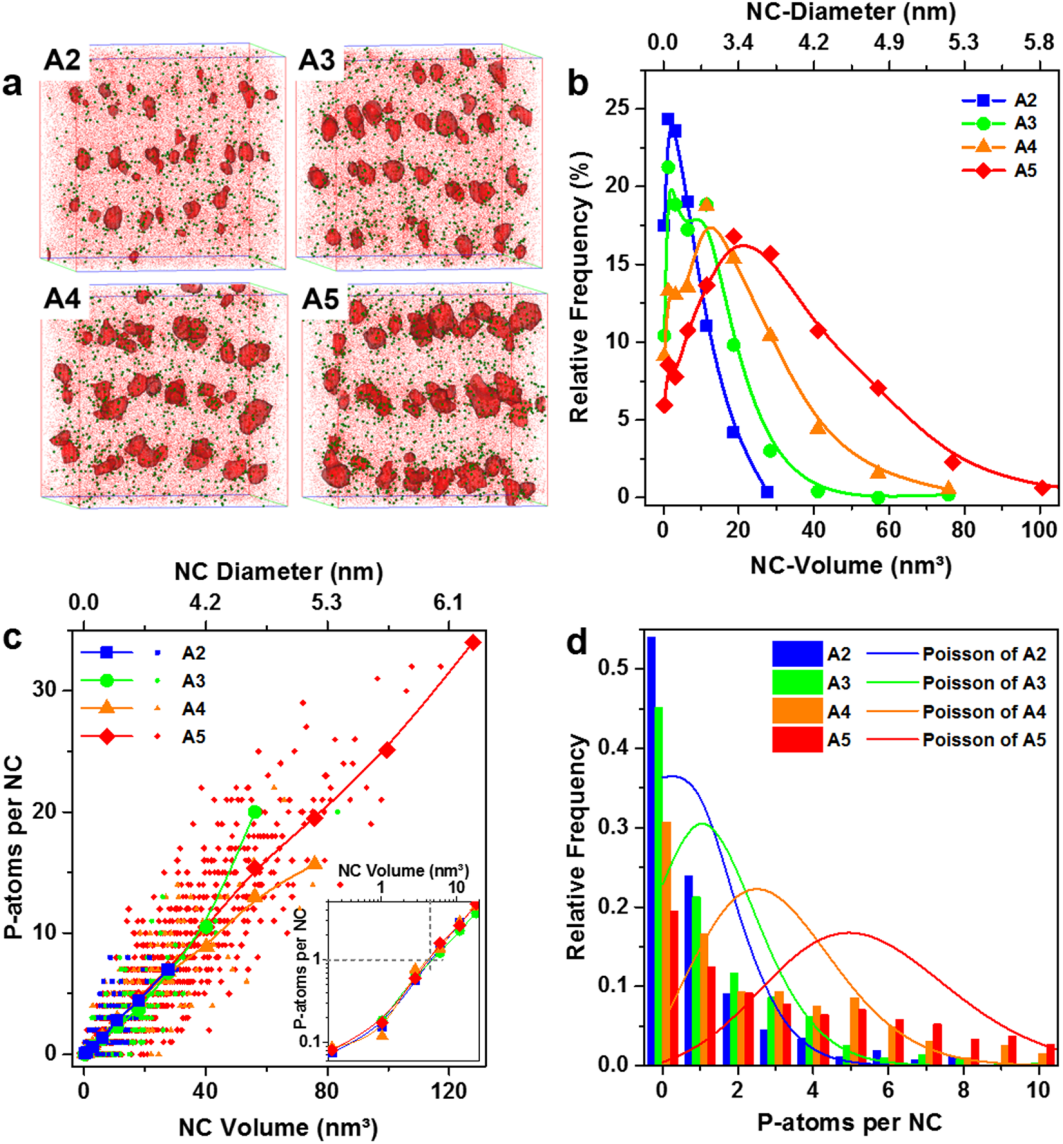
Atom probe tomography analysis. (**a**) APT-reconstructions with Si NCs depicted as red Si iso-concentration surfaces (≥70 at%), Si-atoms in red and P-atoms in green. (**b**) Frequency distribution of the Si NC volumes of each sample, the solid lines (splines) are just a guide to the eye. (**c**) The number of P-atoms per NC as function of NC volume represents an approximately linear behaviour (small symbols: APT raw data, large symbols: average values from grouping NCs in volume classes). The inset magnifies the region around one P-atom per NC. Grey dashed lines show that NCs with 1 P-atom are on average at d_NC_ ≈ 2 nm. (**d**) Comparison of the relative frequencies of NCs with *n* P-atoms per NC as measured by APT (bar graph) and ideal Poisson distributions with the average number of P-atoms per NC in each sample as expected value (solid lines). Due to the NC size dispersion the distributions are clearly non-Poissonian but they can be fitted as exponential probability distributions (fits not shown for the sake of clarity).

APT allows for the determination of the Si NC size distribution and the distribution of P-atoms as function of NC size. For this analysis, we define NCs by iso-concentration surfaces of ≥70 at% Si. Reconstructions of all A-samples are shown in Fig. 1a. The true number of P-atoms inside of NCs is affected by two artefacts, namely local magnification effects (LME)^16, 22–24^ and the limited detection efficiency of APT. As explained in Supplementary Fig. S2, both artefacts are counteracting and although they do not fully compensate each other, we only use the as-measured APT data for further analysis. In proximity histograms (Fig. S2) the P-concentrations inside NCs seem to be rather constant as shown recently for various sample fabrication methods^25^. A determination of a possible preferential location of the P-atoms within the Si NCs is beyond our study, though a preferential location at ~5 Å under NC surface was predicted in theory^26^. In Fig. 1b the relative frequency of the Si NC volumes and the corresponding NC diameters for each A-sample are shown. Clearly, the samples have different mean sizes and all distributions are log-normal with a pronounced tail towards NC volumes or sizes significantly larger than the respective mean size. The mean sizes and the areal NC densities per layer derived from APT analysis are summarized in Table 1.

Next, we analyse the distribution of P-atoms over the different NC sizes within each sample. Therefore, the NC ensemble is grouped in volume classes (in steps of 0.5 nm diameter) and the number of P-atoms and NCs in each group is determined from the APT data. Figure 1c plots the respective ratio, i.e., the average number of P-atoms per NC as function of its volume (large symbols), together with the APT raw data (small symbols). Surprisingly, it turns out that all samples feature a similar distribution irrespective of the mean NC size. Moreover, there is an almost linear dependence of the NC-volume and the average number of P-atoms, so that solely the size of an individual NC determines its probability of being host of a single or multiple P-atoms (the slope is ~0.25 P-atoms per nm³). Specifically, NCs with ~1 P-atom are in all samples ~4.5 nm³ large, which equals a diameter of ~2 nm (grey dashed lines in the inset of Fig. 1c). On first sight this behaviour seems to contradict the commonly assumed Poisson distribution of P-atoms^27, 28^. However, a Poisson distribution is only valid for monodisperse NC ensembles with identical size. In order to illustrate the deviation from Poissonian P-distributions due to the NC size distribution, we plot the relative frequencies of NCs with *n* P-atoms together with ideal Poisson functions in Fig. 1d using the average number of P-atoms per NC (cf. Table 1) as expected value. Obviously, both distributions have nothing in common. In fact, the distributions shown as bar graphs in Fig. 1d can be fitted as exponential probability distributions *f*(*x*) = *λ*·*e* ^*λx*^, where the parameter *λ* is the inverse of the expected value, which equals indeed approximately the average number of P-atoms per NC ([P]_APT_ in Table 1). We conclude that within each sample or NC-ensemble the smallest NCs have the highest probability of being P-free, whereas the largest sizes of the ensemble will incorporate several P-atoms. Self-purification^20^ implicates that the P-concentration (i.e., number of P-atoms divided by all Si-atoms in the NC) decreases with decreasing NC size. As shown in Supplementary Fig. S3, this trend cannot be observed in our data set, indicating that self-purification effects do not dominate the Si NC:P/SiO_2_ system. On average, the P-concentration of sample set A is ~3 × 10^20^ cm^−3^, i.e., slightly below the solubility limit of P in bulk-Si (4 × 10^20^ cm^−3^ at 1150 °C annealing temperature; cf. ref. 13). In particular, 15% (A5) to 35% (A2) of the NCs exceed the bulk solubility limit (cf. Fig. S3).

### Photoluminescence quenching by P-incorporation into Si NCs

**Figure 2:**
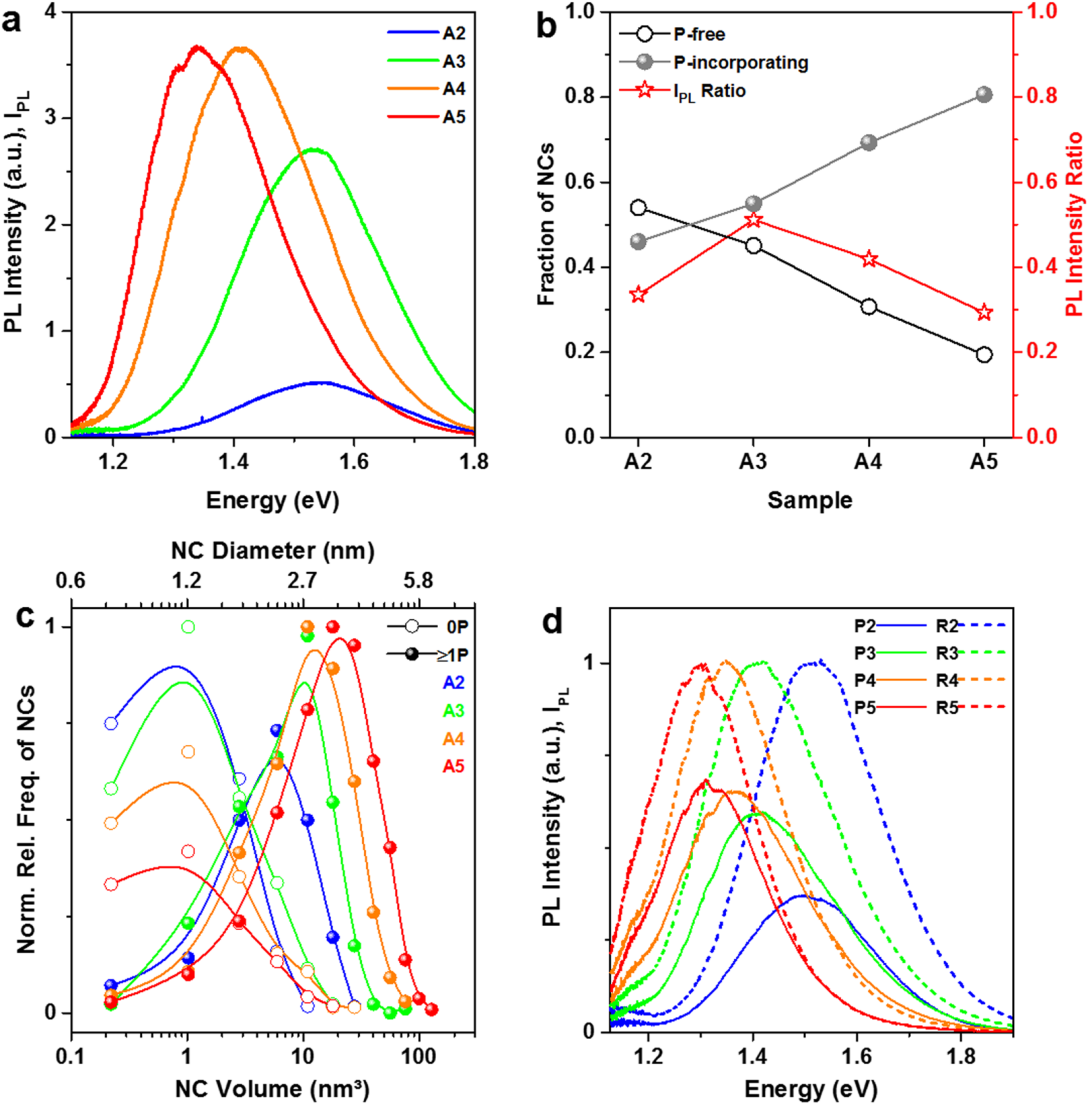
Photoluminescence analysis. (**a**) PL spectra of all A-samples (H_2_-passivated). Despite substantial P-concentrations NC-size dependent PL spectra are measured. (**b**) Comparison of the fraction of P-incorporating and P-free NCs with the integrated PL intensity ratios (P-doped divided by undoped references) of sample set A. Except for A2, the PL intensity seems to scale with the amount of undoped NCs in each sample. (**c**) Size distribution of the P-free (open symbols) and P-incorporating (filled symbols) subsets of the NC ensemble. The P-free NCs in each sample have a mean diameter of ~1.3 nm, while the size distribution of the P-incorporating NCs follows the trend of SRON:P thickness. (**d**) PL spectra of all H_2_-passivated samples of set E. Each spectrum of the undoped reference samples (dashed lines) is normalized to 1 and the respective P-doped sample (solid lines) is scaled by the same factor. The P-doped SiO_2_ reference without Si NCs (sample SiO_2_:P) does not emit any measurable PL and is therefore omitted here.

According to literature, P has two effects on Si NCs: The passivation of some surface defects (PL intensity increase)^27, 29–31^ and the non-radiative recombination via P-induced free carriers (PL intensity decrease)^1, 31, 32^. The dangling bond passivation by P-atoms is qualitatively not comparable to standard H_2_ passivation, which inactivates virtually all defects^33, 34^. We annealed all samples in H_2_ (450 °C, 1 h) so that only PL-quenching by P is visible. Under low excitation power density (≪1 Wcm^−2^)^35^ the PL intensity is a linear function of the number of luminescent NCs in the each sample. Figure 2a shows PL spectra of sample set A, demonstrating that intense PL is still measureable despite heavy P-doping (~0.6 at%). Assuming that every P-atom inside a NC generates a free carrier capable of non-radiative Auger recombination, all P-free NCs are PL-active and all the P-incorporating NCs are dark. Indeed, the PL intensity ratios of P-doped and P-free samples (39% on average over all samples) roughly corresponds to the fraction of P-free Si NCs (37% on average over all samples). As shown in Fig. 2b, this relation is particularly pronounced for the samples with larger NC mean sizes (A4, A5). However, the PL peaks show a clear shift towards lower emission energies with increasing average NC-size, although large NCs without incorporated P-atoms are very rare. To underline this discrepancy we plot the size distributions of P-free and P-incorporating NC subsets in Fig. 2c. While for all samples the smallest NCs with a mean diameter ~1.3 nm dominate the P-free NC subset, the size distribution of the P-incorporating NCs follows the trend of SRON:P thickness, cf. Fig. 1b and Table 1. Mean sizes of the P-incorporating NC subset are 2.6, 3.1, 3.5, 4.0 nm for samples A2 to A5, respectively, and these NCs emit PL efficiently. Otherwise, all PL spectra of sample set A would have a similar PL peak energy corresponding to the smallest (P-free) NCs. For instance, even NCs of 4–5 nm in size which incorporate several P-atoms (>10, cf. Fig. 1c) have to emit PL to create the PL spectrum of sample A5. Since the PL spectra apparently correspond to the whole NC size distribution, we conclude that only a minor fraction of all P-atoms inside NCs quench PL. This finding challenges the concept of Auger recombination by P-induced free carriers.

Figure 2d shows the PL spectra of sample set E, i.e., samples with lower P-concentrations that equal on average ~1 P-atom per NC (P2 to P5) and the corresponding undoped reference samples (R2 to R5). At moderate doping levels, we observe a clear size-dependence of the PL quenching, while the peak energy does not shift significantly by P-doping. The smaller the mean NC size of the sample, the less PL is emitted although the average P-concentration is ~1 P-atom per NC for all samples, as shown in Supplementary Fig. S4. This correlation appears counterintuitive since the largest NCs (lowest QC) are supposed to allow for most efficient doping, whereas here apparently the smallest NCs are subject to the strongest P-induced PL quenching. In essence, the results presented here suggest that a different mechanism than Auger recombination with P-induced free carriers causes the PL quenching, which will be addressed in the Discussion Section.

### Ionization energy of P-induced majority charge carriers in Si NCs

**Figure 3:**
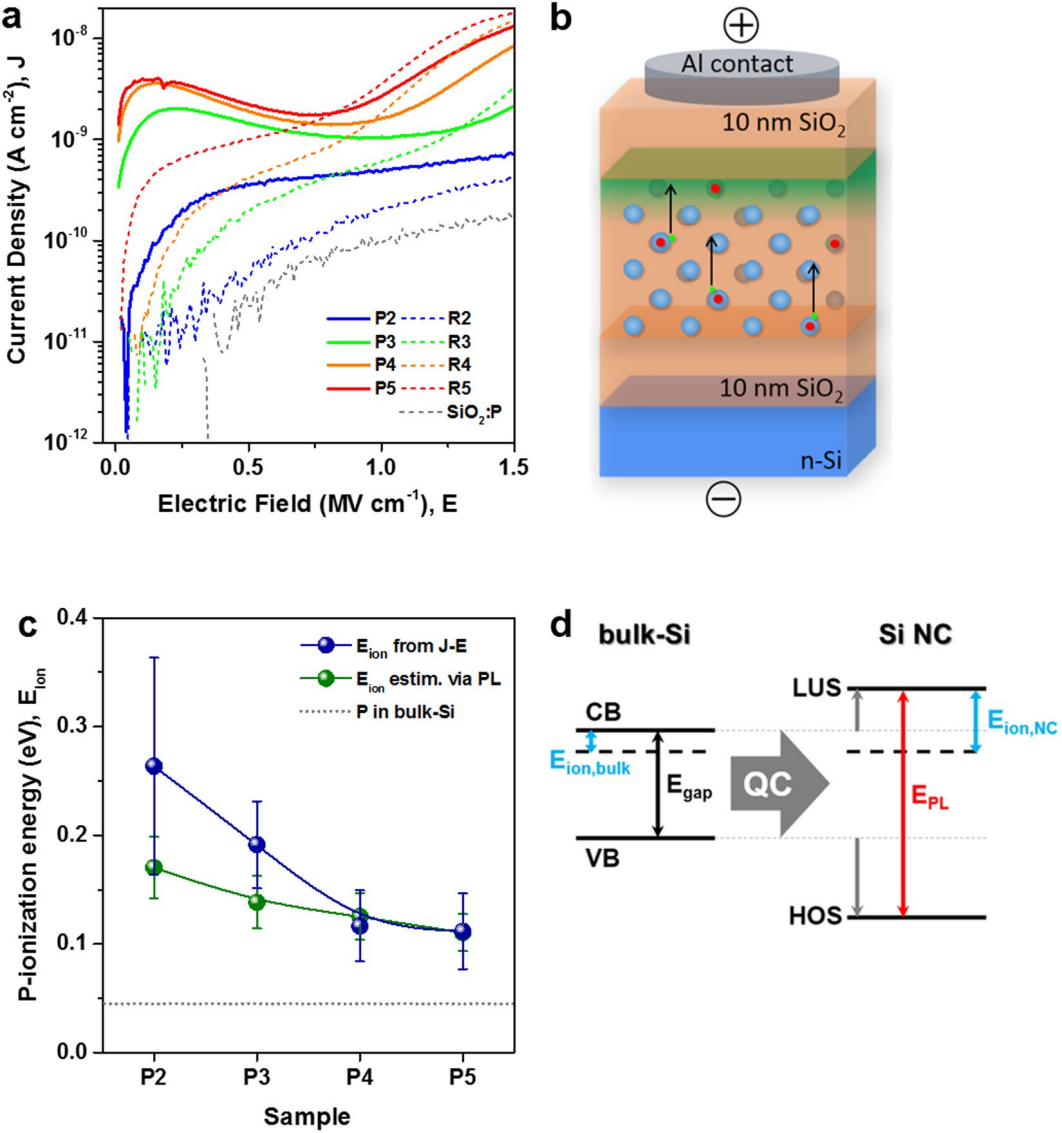
Current-voltage measurements (I-V) and derived ionization energies of P-atoms in Si NCs. (**a**) Current density vs. electric field of sample set E (P: Si NCs incorporating on average ~1 P-atom per NC, R: undoped reference samples). At low E-fields the P-samples (solid lines) clearly exhibit higher displacement current densities than the respective reference sample (dashed lines). (**b**) The schematic shows the sample structure (large blue spheres: Si NCs, red points: P-atoms in Si NCs, small green points: donor electrons localized at P-atoms in thermal equilibrium): Since charge injection from gate or substrate is blocked at low fields, only transient displacement currents caused by P-donors are measured, which accumulate under the gate electrode (shown in green). (**c**) Ionization energies of P-donors derived from I-V measurements according to Equation (2) with the mean size of the P-incorporation fraction of NCs (cf. Fig. 2c) as *d*_*NC*_ (blue); and P-ionization energies estimated via the PL-peak energy (green). Both methods yield similarly high P-ionization energies compared to P in bulk-Si (45 meV; grey dotted line). The values increasingly deviate from each other towards smaller NCs but are still within the error bars, which are based on the standard deviations of the NC-size and the uncertainties of the J-peak or respectively the PL peak width. (**d**) Schematic for the estimation of *E*_*ion*_ via PL, assuming a Si NC bandgap energy equal to the PL peak energy and a QC-induced LUS-to-HOS shift ratio of 1:2 ^37^.

In order to investigate the ionization behaviour of the P-incorporating Si NCs, we used metal-oxide-semiconductor (MOS) capacitors with Al contacts and 10 nm thick SiO_2_ injection barriers to prevent charge injection into the Si NCs from gate or substrate (cf. ref. 36). The number of superlattice layers was adjusted to achieve identical total thicknesses (~100 nm) for all samples. The J-E curves are shown in Fig. 3a. First of all, the reference sample SiO_2_:P (SiO_2_ with 0.19 at% P without Si NCs) does not show any features so that free carriers from P-states in SiO_2_ are ruled out. Secondly, for low electric fields all P-doped samples (solid lines) have much higher current densities than their undoped reference samples (dashed lines). There is a clear J-E peak around 0.2 MV/cm which shifts to higher E-fields with decreasing NC size. For sample P2 this peak is not clearly pronounced anymore but a one order of magnitude increased current compared to sample R2 remains.

We argue that P-atoms located on lattice site positions in Si NCs are ionized due to the electric field and consequently donate electrons. The J-peak originates from donor electrons that hop through the sample in the external electric field towards the gate blocking oxide, as shown schematically in Fig. 3b. This internal charge redistribution induces a displacement current, which is measured in the external circuit. In contrast to bulk-Si, the P-atoms in Si NCs are not significantly ionized by thermal activation at room temperature, as proven recently by X-ray absorption spectroscopy at the P K-edge^4^. Whereas the position of the P-donor state in the Si bandgap is considered constant, the conduction band (CB) edge shifts to lower energies (with respect to vacuum level) due to quantum confinement. As a result, the P-ionization energy increases from 45 meV to values where the thermal energy at room temperature is not sufficient for significant ionization anymore. Here, the E-field provides the energy for ionization and accordingly higher E-fields are required for maximum P-ionization in smaller Si NCs with stronger QC. Further considerations about the J-E behaviour of all samples up to the onset of Fowler-Nordheim tunnelling as well as a P2.5 sample that clarifies the transition from the clear J-peak of sample P3 to the levelled J-feature of P2 are presented in Supplementary Fig. S5. From the electric field of the J-peak (***E***_*peak*_, cf. Table 1) we can derive the activation energy (*E*_*ion*_) of the P-ionization process. Therefore, we assume a capacitive voltage divider between Si and SiO_2_ and consider that the voltage drops only over the SiO_2_ layers with a total thickness of *t*_Σ*SiO*2_:

1 $${E}_{ion}={{\boldsymbol{E}}}_{peak}\cdot \frac{{t}_{total}}{{t}_{{\rm{\Sigma }}SiO2}}{d}_{NC}\cdot e,$$

where *d*_*NC*_ denotes the NC-size, ***E***_*peak*_ the electric field of the J-peak (in units of V/m), *t*_*total*_ the total superlattice thickness and *e* the elementary charge. The *E*_*ion*_ values of the P-ionization energy using the mean size of the P-incorporating fraction of NCs (cf. Fig. 2c) range from ~110 to ~260 meV and are shown in blue in Fig. 3c. To put these ionization energies in perspective, we consider the PL peak energies as a manifestation of the Si NC fundamental gap. By means of the bulk-Si bandgap at room temperature (1.12 eV) and a quantum confinement induced shift ratio of 1:2 of the lowest unoccupied states (LUS, equivalent to the CB edge in the bulk) and the highest occupied states (HOS, equivalent to the valence band (VB) edge in the bulk), cf. ref. 37, the LUS-energy for each sample is estimated. Adding the bulk-Si P-ionization energy of 45 meV gives the total P-ionization energy for P in Si NCs. The schematic in Fig. 3d illustrates this consideration. The resulting ionization energies (green in Fig. 3c) range from ~110 to ~170 meV. Due to the NC size distribution the error bars of the *E*_*ion*_ values measured by I-V are rather large, especially for sample P2, but still we can conclude that both methods are in accord with each other. Besides quantum confinement, also dielectric confinement increases the ionization energies when the donor wavefunction is not fully screened by surrounding silicon^38^, i.e., predominantly for the smallest NCs. Generally, we note that due to ionization energies as derived from I-V measurements of $\sim 2.4\ldots 5.8\times {E}_{ion,bulk-Si}$, the ionization probability $(\propto {e}^{-\frac{{E}_{ion}}{{k}_{B}T}})$ inherently drops to values on the order of 0.01…1%, leaving the vast majority of P-atoms in NCs unionized.

### Doping efficiency and formation energy of P-doped Si NCs

**Figure 4:**
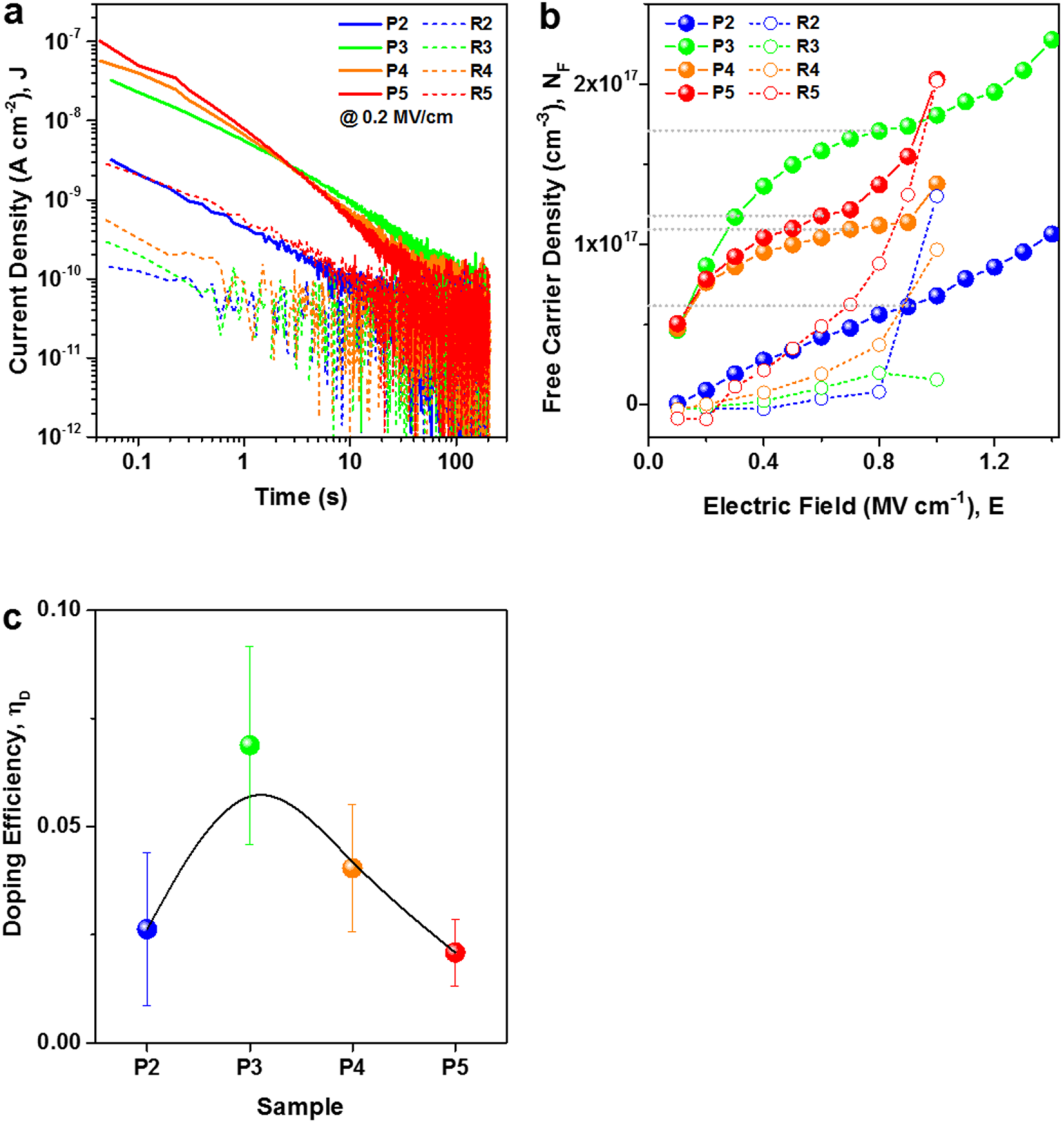
Free carrier density and doping efficiency. (**a**) J-t-measurement of all samples at an E-field of 0.2 MV/cm. The transient currents scale within one order of magnitude with the increasing Si-content of the sample and are increased by up to two orders of magnitude by P-doping. (**b**) Density of charge carriers *N*_*F*_ as function of electric field, measured at room temperature. Each data point represents a J-t measurement on a virgin contact, so that any influence from charging of a preceding measurement is avoided. The grey dotted lines indicate approximately the saturation point. For *η*_*D*_ calculations, the background carrier density of the undoped reference samples at saturation is subtracted. (**c**) Doping efficiency *η*_*D*_ as ratio of free carriers (at E-fields of ~0.1 to 0.6 MV/cm) and the number of P-atoms incorporated by Si NCs. The black solid line (spline) is just a guide to the eye.

Besides ionization energy, the second figure of merit in electronic doping is the doping efficiency, i.e., the number of free charge carriers per P-atom in a Si NC. We focus here on the charge carriers that are generated by the electric field in the MOS capacitor. These carriers are not *free* in the classical sense (at room temperature and in the absence of an electric field) but they can be released from their parent donor atoms by field emission. In order to measure the total amount of mobile charges, the current density at a fixed E-field is measured over time (J-t-measurement). Since the SiO_2_ injection barriers prevent injection of carriers from the terminals of the MOS capacitor up to the onset of Fowler-Nordheim tunnelling, only transient currents are measured. J-t curves exemplarily measured at an E-field of 0.2 MV/cm are shown in Fig. 4a. Clearly, there is a higher transient current in the P-samples compared to their undoped references (R-samples). For ~100 s the currents of all samples approach zero (sub-pA level). This transient current originates from an internal charge redistribution of electrons accumulating under the gate electrode (cf. schematic in Fig. 3b). Similar to the concept of image charges, this redistribution induces a current in the external circuit which is measured as *J*(*t*). The amount of mobile or *free* charges *N*_*F*_ is determined via the Shockley-Ramo theorem^39–41^:

2 $${N}_{F}=\frac{1}{e\,\cdot \,{x}_{mean}}\int J(t)\,dt,$$

where *e* denotes the elementary charge and *x*_*mean*_ the average distance the carriers have to travel during the charge redistribution process (here half of the total superlattice thickness, i.e., ~40 nm). Figure 4b shows the *N*_*F*_ values of all samples as function of E. For samples P3 to P5 we observe a saturation behaviour at carrier densities in the low 10^17^ cm^−3^ range. Further increase of *N*_*F*_ values indicates the onset of other charge generation mechanisms as explained in Supplementary Fig. S5. As before, the saturation is hardly visible for sample P2. The saturation point indicates that all P-donors capable of providing an electron are ionized by the electric field. The undoped reference samples show about one order of magnitude lower *N*_*F*_ values at low E-fields. In order to consider only charges clearly associated to P-doping, the values of the respective reference samples at the saturation point are subtracted: *N*_*F,eff*_ = *N*_*F,P*_−*N*_*F,R*_. The denominator of the efficiency calculation is the total number of P-atoms incorporated by NCs. In the MOS capacitors it is simply given by the number of NC-layers in the SL *N*_*SL*_, the NC areal density ρ_NC_ (from APT measurements, see Table 1), and the average number of P-atoms per NC ${[P]}_{APT}$. The doping efficiency of incorporated P-atoms *η*_*D*_ is then calculated by:

3 $${\eta }_{D}=\frac{{N}_{F,eff}}{{N}_{SL}\,\cdot {{\rm{\rho }}}_{NC}\,\cdot \,{[P]}_{APT}}.$$

Figure 4c shows the doping efficiency values. On average, over all samples only ~3.9% of the P-atoms embedded in Si NCs contribute one electron, when field emission in the electric field takes place. The doping efficiencies increase with decreasing average NC-size and reach the highest value of ~7% for sample P3 before dropping again for P2 (which is the most difficult sample as discussed above). Larger free carrier densities and doping efficiencies for smaller NCs appear counterintuitive. However, a glance at the ratio of NC-internal bonds per Si-atom for such small Si NCs reveals values significantly below the bulk limit of 2 bonds/atom^42^. Thus, a possible cause for the increasing *η*_*D*_ could be the inability of small NCs to provide enough counter-stress for preventing P to be incorporated substitutionally on a lattice site.

## Discussion

The optical and electrical results presented so far are inconsistent with respect to the role of P-atoms incorporated in Si NCs. The PL quenching by P-atoms in small Si NCs is more efficient than in larger NCs (cf. Figs 2b and S4). Hence, at room temperature and in the absence of any electrical field the PL of large NCs is less influenced by incorporated P-atoms, although larger NCs generally incorporate more P-atoms than smaller NCs (cf. Fig. 1c). On average, P-atoms quench ~40% of the luminescence. In contrast, the analysis of the electrical measurements revealed P-ionization energies of ~110–260 meV. Such energies do not allow for significant free carrier generation to trigger Auger recombination to a magnitude where the associated PL quenching can be detected. Besides, the ionization energy increases with decreasing NC size, while the PL quenching activity decreases. Hence, the optical and the electrical influence of P on nanocrystalline Si have a different NC size dependence and orders of magnitude different efficiency. This result implicates that both effects must have a completely different origin. The infinitesimal amount of free electrons from P-doping can neither explain the PL quenching nor its NC size-dependence. Therefore, the classical concept of PL quenching via Auger recombination of the exciton with a donor electron is rendered invalid.

**Figure 5:**
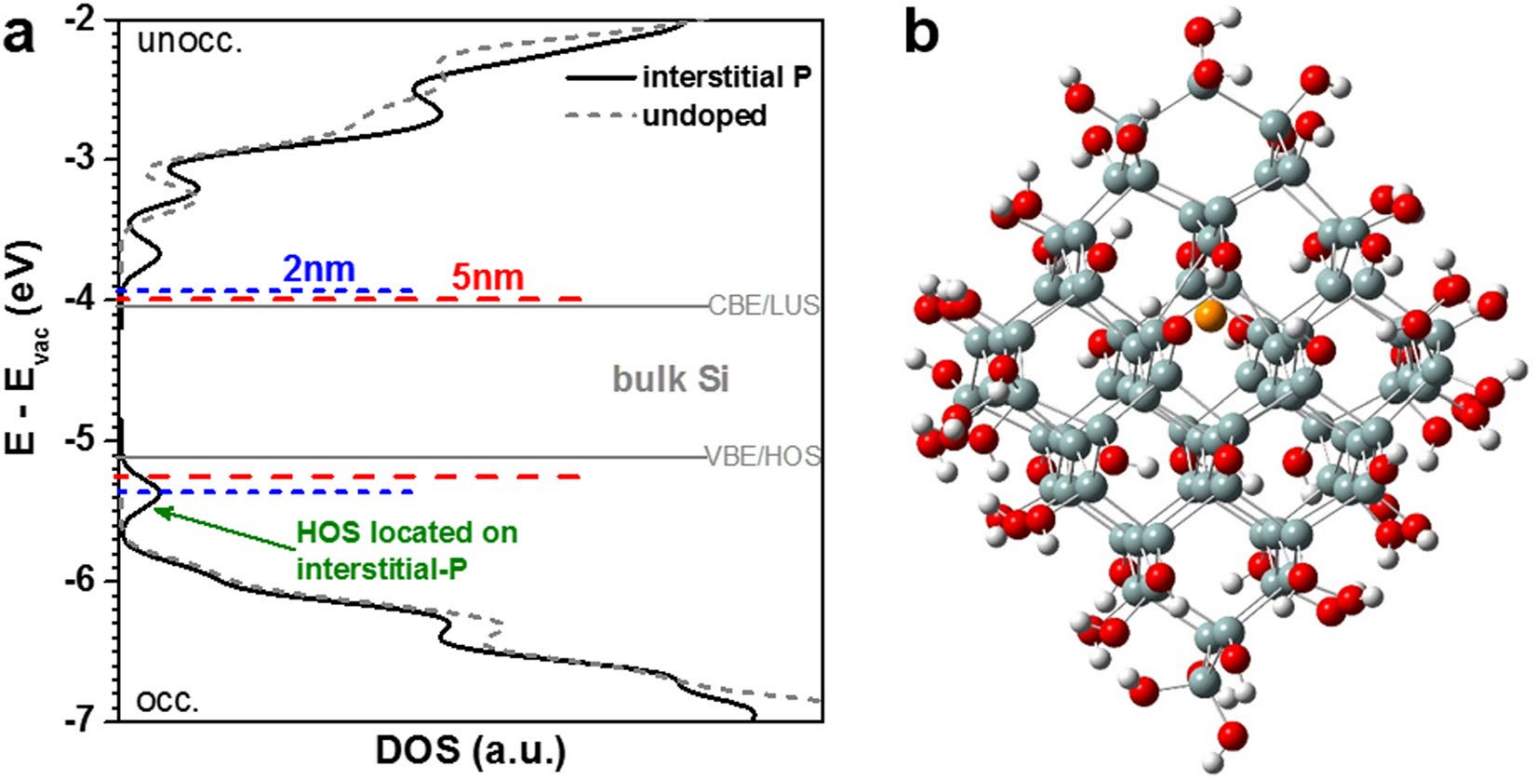
Results of density functional theory simulations. (**a**) Density of states (DOS) of a pristine Si_84_(OH)_64_ approximant (grey dotted curves, d_NC_ = 1.5 nm) together with the DOS of a Si_84_(OH)_64_ approximant containing interstitial P (black solid curves). The latter is shown in (**b**) with an interstitial P-atom in the NC centre (Si-atoms: grey, O-atoms: red, H-atoms: white, P-atom: orange). For bulk Si (grey solid lines) the HOS-level of interstitial P is well below the VB-edge and hence no recombination centre. QC-induced widening of the fundamental gap results in the HOS energy of Si NCs approaching the P-induced defect level. As a consequence, small NCs (blue dotted line) are more strongly subject to PL quenching than larger NCs (red dashed line).

By means of density functional theory (DFT) we demonstrated recently that P-atoms located in the Si/SiO_2_ transition shell on the surface of a NC or on interstitial lattice sites generate states in the fundamental gap of Si NCs^4^. Whereas both of these states could act as PL quenching centres, the interstitial-P configuration is of special interest, since its HOS overlap only for the smallest Si NCs with the HOS of the Si NC (equivalent to the VB edge in the bulk). Larger Si NCs with less quantum confinement merely touch this level. The idea that the P-induced PL quenching centre is located in the NC volume and not in the sub-oxide shell around it is further supported by PL measurements on freestanding H-terminated Si NC, which show the same trend^43^. The energetic situation is depicted in Fig. 5a, where we identify again the energy of the fundamental Si NC gap with the PL peak energy. As above, we consider the QC-induced LUS-to-HOS shift ratio as 1:2 ^37^.

Following our concept, a certain redshift of the PL peak is expected for P-incorporating NC samples, since preferentially the smallest NCs of each ensemble are quenched by the interstitial-P induced state. Indeed, a very small PL redshift of ~15 meV is observed for sample P2 compared to R2 (see Fig. 2). However, while for the samples with larger NC mean sizes (i.e., only a small fraction of small NCs) a vanishing redshift is expected, we even observe a small blueshift of the PL peak. It cannot be ruled out that also other effects play a role here, such as a slightly different Si NC growth due to the presence of P (decreased oxide viscosity).

**Figure 6:**
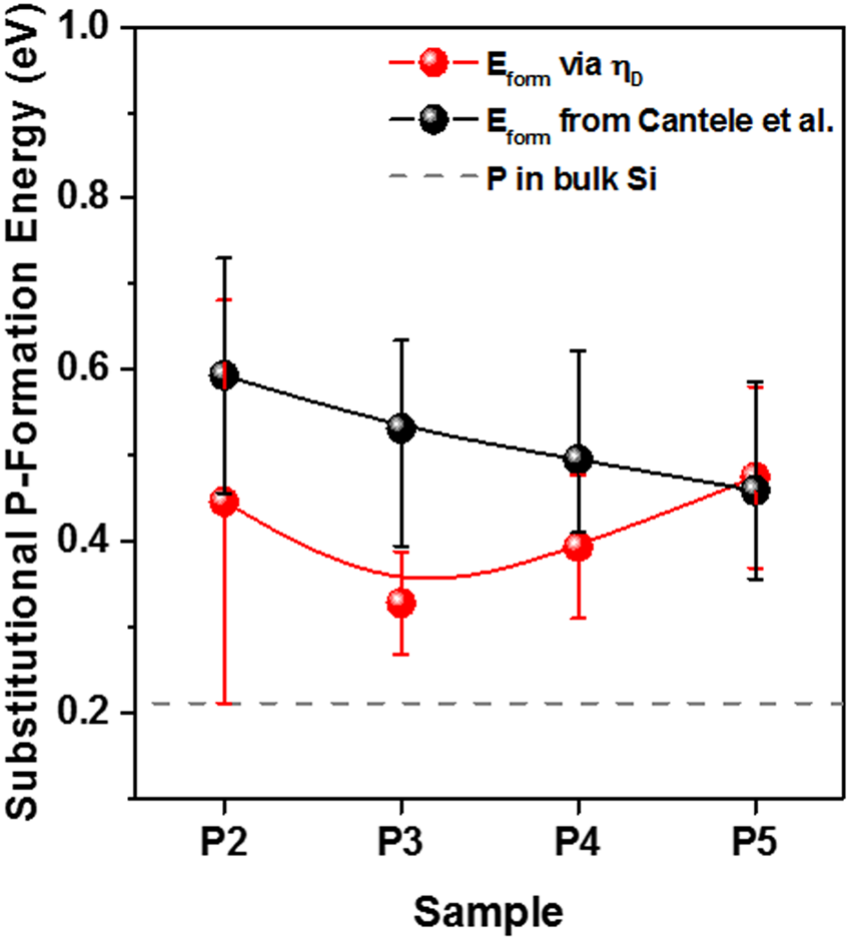
Formation energies of substitutional P-atoms in Si NCs. The results from Equation (4) using the doping efficiency are shown in red. The formation energies calculated using the results presented in ref. 44, i.e., ${E}_{form}=0.21008+4.98131/(0.5\,{d}_{NC})$ with energies in units of eV and NC-radii in Å, are shown in black. The grey dashed line represents the bulk formation energy^44^. The solid lines (splines) are just a guide to the eye.

Interstitial P, P-atoms on the Si NC-surface, or SiO_2_:P configurations in the vicinity of a NC do not provide an occupied state near the LUS (equivalent to CB in the bulk)^4^. The fact that we measure free charge carriers by I-V with rather high ionization energies as a result of quantum confinement (cf. Fig. 3c) suggests that actual P-donors are present in the Si NCs. This in turn allows interpreting the doping efficiency *η*_*D*_ as the fraction of P-atoms on Si lattice site positions, since only in this configuration electronic doping is possible. The formation energy *E*_*form*_ of a P-atom on a Si-lattice site position in the NC is determined via:

4 $${E}_{form}=-{k}_{B}{T}_{a}\cdot \,\mathrm{ln}\,{\eta }_{D},$$

where *T*_*a*_ denotes the annealing temperature at which the P-doped Si NCs were formed (here 1423 K). As shown in Fig. 6 (red symbols) the formation energies derived in that way are on average ~410 meV, i.e., about twice the formation energy in bulk Si. For comparison, we also plot in Fig. 6 (black symbols) the theoretically calculated formation energies for P-atoms on Si-lattice site positions presented in ref. 44. The experimental values range mainly at the lower end of the error bars of the theoretical values, though it has to be mentioned that Cantele *et al*.^44^ only considered very small (0.52–1.12 nm radius) and approximants with full H-termination. Irrespective of the experimental errors and the different theoretically studied sample system, Si NCs have significantly higher substitutional P-formation energies as compared to bulk-Si. Accordingly, only a small percentage of all incorporated P-atoms resides on Si lattice sites where they can become donors by field emission, leaving the vast majority of P-atoms on interstitial sites. As discussed before, the trend of decreasing *E*_*form*_ for samples P5 to P3 might originate from the insufficient number of NC-internal bonds per Si-atom^42^ that exert counter-stress against a substitutional P-incorporation. Indications of an increased doping probability of ultra-small NCs exist^4, 45^ though characterization or computational treatment of this phenomenon is currently beyond state-of-the-art technical capabilities.

Finally, the role of multiple P-donors in one NC is addressed. Although unlikely at the moderate doping levels used here, it is possible to find more than one substitutional P-atom in one Si NC, in particular for the largest NCs (d_NC_ ≈ 5 nm). In this configuration, the ionization is further complicated by exchange interactions. The exchange interaction influences the energy levels of both P-atoms in a non-trivial way since the exact atomic configuration of the P-dimer determines the exchange energy, as investigated in detail by Pereira *et al*.^46, 47^. Although this issue is not considered in detail here, we argue that low free carrier densities caused by small amounts of substitutional P-atoms cannot be efficiently overcome by increasing P-concentrations. While higher P-concentrations increase the number of P-incorporating NCs and substitutional P, the number of NCs with multiple substitutional P-atoms also increases, which does not simply increase *N*_*F*_ due to exchange interaction.

## Conclusion

P-atoms incorporated in Si NCs have a fundamentally different impact on the optical and electrical properties. Two different P-configurations, interstitial and substitutional P, allow for luminescence quenching and non-thermal charge carrier generation, respectively. In detail:

(i) Free majority charge carriers from P-donors do not cause PL quenching. Instead of Auger recombination, we propose non-radiative recombination via states induced by interstitial P-atoms as its origin. In general, the bare observation of luminescence quenching is no evidence for actual electronic doping.
(ii) In the presence of an electrical field, P-incorporating NCs can provide charge carriers that originate from actual P-donors (substitutional P). Depending on the NC size the E-field has to overcome the P-ionization energies of ~110 to ~260 meV. Without E-field there are no measureable P-induced free carriers in Si NCs since the thermal energy at room temperature (*k*_*B*_ *T* ≈ 25 *meV*) is not sufficient for significant ionization.
(iii) Merely ~4% of the P-atoms incorporated in NCs provide charge carriers under field emission with E ≈ 0.1–0.6 MV/cm. This doping efficiency is interpreted as the fraction of P-atoms on substitutional lattice sites, which corresponds to formation energies of ~400 meV.

In short, classical electronic P-impurity doping of ultrasmall Si nanovolumes is not feasible in an efficient manner. We emphasized that the inability of efficient P-doping is of fundamental physical nature (increased *E*_*form*_ due to small crystal volumes and increased *E*_*ion*_ due to quantum confinement). Hence, there is no technological or engineering solution. While it is difficult to extrapolate from the presented data at which size the doping efficiency is not affected anymore, we can exclude useful P-doping for Si volumes <100 nm³ or ~6 nm diameter. Future CMOS technology nodes target these dimensions though limitations in device performance and reliability related to inefficient doping are expected to occur already at larger dimensions. Recently, we proposed an alternative doping technology based on Al-induced acceptor states in Si-adjacent SiO_2_ that capture an electron from the Si VB, leaving a hole as free majority carrier behind^48^. Since this modulation doping approach relocates the impurity dopants from Si into SiO_2_, the nano-size effects mentioned above do not limit doping efficiencies.

## Methods

### Sample fabrication

Si NC superlattices were fabricated by PECVD^49^ of alternating SiO_x=0.93_N_y=0.22_ (SRON; 2, 3, 4, 5 nm thick) and SiO_2_ barrier layers on 100-oriented Si wafers. For P-doping 2–10 sccm of 1% PH_3_/Ar were added, depending on the intended P-concentration (cf. Fig. S1 and Table 1). For sample set A (dedicated to APT measurements) 30 bilayers with 5 nm SiO_2_ barriers were deposited on lowly doped p-type Si substrates. For sample set E (dedicated to electrical measurements) 20, 16, 14, 12 bilayers for 2, 3, 4, 5 nm SRON, respectively, were deposited so that the total thickness of each SL is ~80 nm. The SiO_2_ barriers were always 2 nm thick and the films were deposited on n-type (1–30 Ω cm) Si substrates. In addition to P-doped samples (labelled P-*SRON thickness*), sample set E also contains structurally identical undoped references samples labelled R-*SRON thickness*. All SLs were sandwiched between 10 nm thick SiO_2_ buffer and capping layers which also serve as electrical injection barriers. After deposition, the samples were annealed in a quartz glass tube furnace at 1150 °C for 1 h in high-purity N_2_ ambient and subsequently defect passivated at 450 °C for 1 h in pure H_2_ ambient. Aluminium contacts were thermally evaporated and photolithographically structured to form MOS-capacitors for electrical characterization.

### Sample characterization

PL was measured using a LN_2_-cooled CCD camera attached to a single grating monochromator and under excitation of a HeCd laser (325 nm line) with a power density of ~3 mW/cm². I-V and I-t was measured in accumulation regime using an Agilent B1500A semiconductor device analyser. The MOS-capacitors were contacted by W-needles in a Cascade M150 Prober located in a shielded darkbox. Needle-shaped tips for APT were structured using an Auriga focused ion beam scanning electron microscope (FIB-SEM, Zeiss) and vertically attached onto the apex of a Mo support grid. For APT measurements a LEAP™ 4000X Si (Cameca) with a pulsed UV laser (355 nm, 100 pJ, 250 kHz), a cooled specimen holder (~40 K), and a chamber pressure of 10^−12^–10^−11^ Torr was used. Data reconstruction was carried out using IVAS^TM^ software (version 3.6.6).

### Hybrid density functional theory (h-DFT) calculations

Approximants were calculated with non-periodic boundary conditions and underwent geometrical optimization with the B3LYP h-DF^50, 51^ and the 6–31 G(d) all-electron molecular-orbital basis set (MO-BS)^52, 53^ using the Gaussian 09 suite^54^. RMS and peak force convergence limits were 15.4 meV/Å (5.67 × 10^−4^ Ha/Å) and 23.1 meV/Å (8.51 × 10^−4^ Ha/Å), respectively. Electronic structures were computed with the 6–31 G(d) all-electron MO-BS with diffuse MO functions^55^ −6-31 + G(d) – to account for the more delocalized nature of shallow states induced by P, resulting in the compute route B3LYP/6-31 G(d) //B3LYP/6-31 + G(d). Deviations from B3LYP/6-31 G(d) //B3LYP/6-31 G(d) calculations^4^ were found to be minute. Additional information is available on accuracy tests and tests of functional group termination as approximation of the dielectric^56–62^. During all calculations, no MO symmetry constraints were applied and tight convergence criteria (≤ 10^−9^) were set for the self-consistent field routine.

## Electronic supplementary material

revised Supplementary Information for production
