# Supplementary material for: Defect-Induced Luminescence Quenching vs. Charge Carrier Generation of Phosphorus Incorporated in Silicon Nanocrystals as Function of Size
Source: Sci Rep. 2017 Apr 13;7:863. doi: 10.1038/s41598-017-01001-1 (PMC5429832; doi:10.1038/s41598-017-01001-1)
Supplement: Supplementary file 1 — revised Supplementary Information for production [file 41598_2017_1001_MOESM1_ESM.pdf]

Supplementary Information

Defect-Induced Luminescence Quenching vs. Charge Carrier Generation of Phosphorus Incorporated in Silicon Nanocrystals as Function of Size

Daniel Hiller*[1], Julian López-Vidrier[1], Sebastian Gutsch[1], Margit Zacharias[1], Keita Nomoto[2, 3] & Dirk König[4]

[1] *Laboratory for Nanotechnology, Dept. of Microsystems Engineering (IMTEK), University of Freiburg, Germany*

[2] *School of Photovoltaic and Renewable Energy Engineering (SPREE), UNSW, Sydney, Australia*

[3] *Australian Centre for Microscopy and Microanalysis, The University of Sydney, NSW 2006, Australia*

[4] *Integrated Materials Design Centre (IMDC), UNSW, Sydney, Australia*

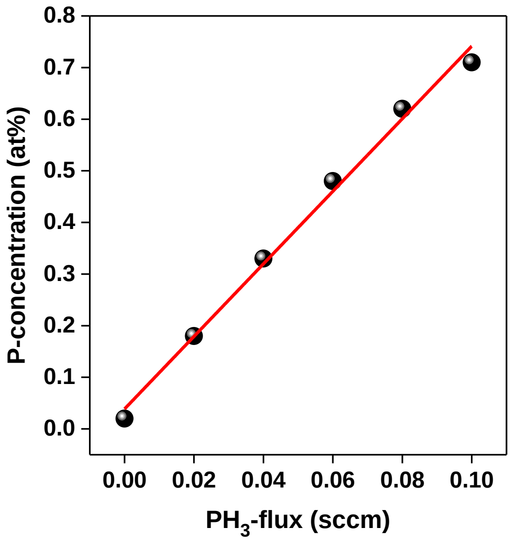

Supplementary Figure S1: P-concentration in $SiO_{x=0.93}N_{y=0.22}$ measured by molecular-MCs^+ secondary ion mass spectrometry (MCs^+-SIMS) as function of PH_3 flux[36]. The red line represents a linear fit. This linear relationship allows for the determination of the required PH_3 flux for a given P-concentration in SRON:P.

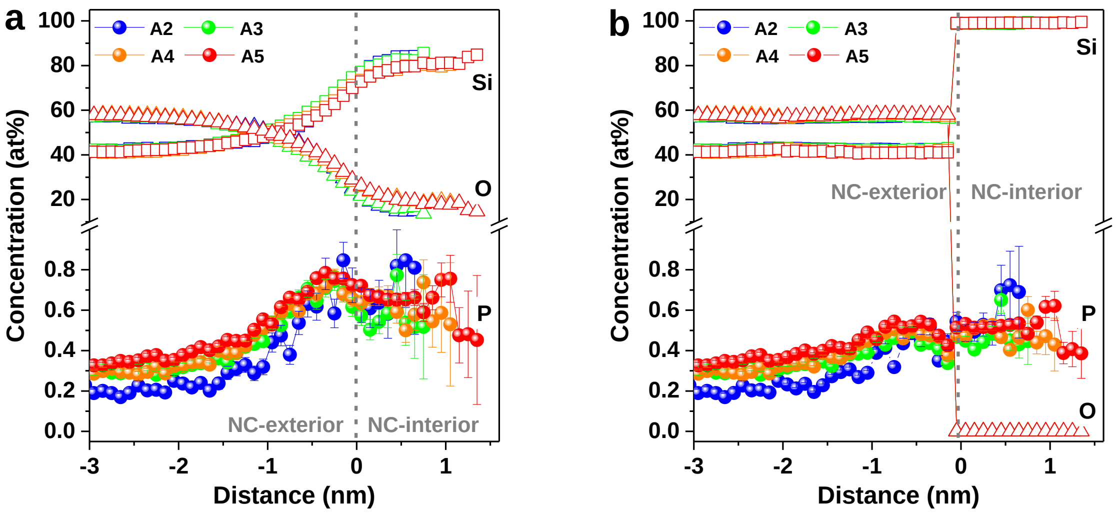

Supplementary Figure S2: (a) Proximity histogram (proxigram[58]) using as measured APT-data. The P-concentration piles up towards the NC interface and has values of 0.6 – 0.7 at% inside of the NCs[17]. We observe that 20 – 30 at% oxygen is projected into the NC-interior (pure Si) by local magnification effects (LME) artefacts. A heterogeneous material system like Si NC/SiO_2 is generally subject LME-artefacts, which leads to the projection of some matrix atoms (O, P) into the NCs. Hence, Si NCs typically do not appear as 100% Si in APT and some P-atoms originally located in a shell around the NCs might be projected into their interior. However, the fraction of O-atoms inside NCs (~20 – 30 at%) allows for a correction of the P-concentration. While LME overestimates the real number of P-atoms inside NCs, the limited detection efficiency of APT (here 57%)[17] underestimates it by detecting less P-atoms in total. Since we cannot correct the P-atoms distribution data accurately for these two counteracting effects, we use the as-measured APT data for analysis and accept the remaining errors in counting individual P-atom as inevitable. The Si NC/SiO_2 interface appears blurred over a distance of 1 – 2 nm, which is commonly attributed to the SiO_x transition shell[59-61]. However, the transition shell is supposed to be only few Å thick. We assume that a convolution of LME artefacts and the transition shell itself generates this broad transition region. (b) In order to estimate the magnitude of these P-projection artefacts we assume an abrupt Si/SiO_2 interface with constant Si- and O-concentrations in the NC-exterior and an O-free interior. The P-concentration is corrected proportionally to the O-concentration. After this correction still ~0.5 at% of P are detected inside the Si NCs, irrespective of the size, while the P pile-up at the interface is attenuated. We are aware that this simple correction will not describe the physical reality exactly, but it demonstrates that the LME artefacts change the P-concentrations just marginally, so that within error bars the as-measured APT data can be used for data analyses.

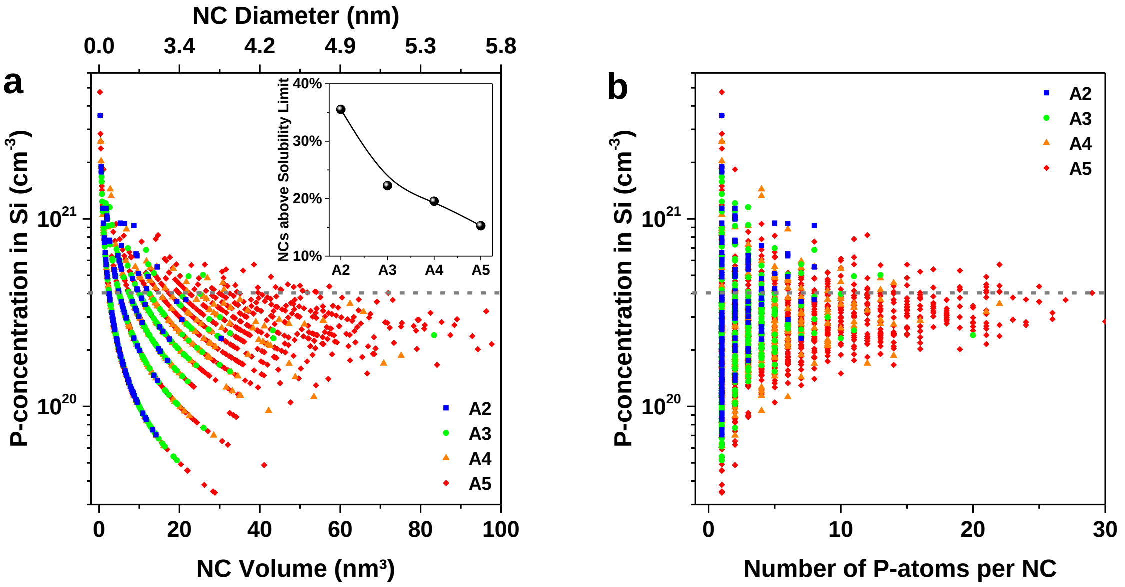

Supplementary Figure S3: (a) P-concentrations as function of NC-size and (b) as function of the amount of P-atoms per NC. The grey dashed lines indicate the solubility limit of P in bulk-Si of 4×10^{20} cm^{-3} for the annealing temperature used here of 1150°C [13]. From both representations it is obvious that smaller NCs and NCs incorporating a single or very few P-atoms have the highest P-concentrations which can exceed the solubility limit. As a possible origin, we identified the low number of bonds between Si NC atoms which can suppress self-purification[42]. The inset of (a) shows that a larger fraction of NCs above the solubility limit is found in samples with a small average NC size.

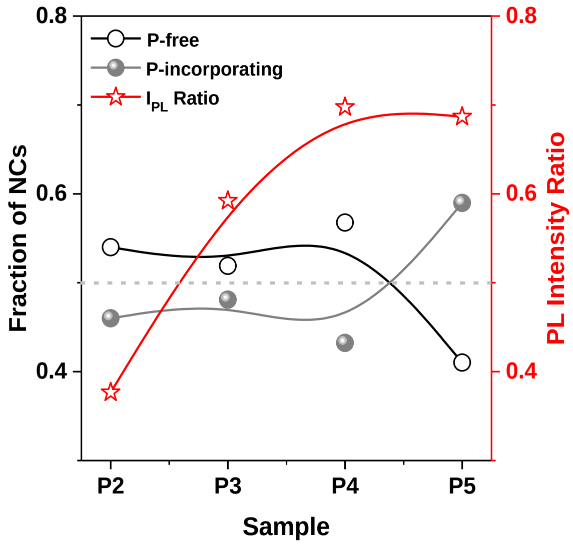

Supplementary Figure S4: Comparison of the fraction (F_P) of P-incorporating and P-free NCs with the integrated PL intensity ratios (P-doped divided by undoped references) of sample set E. For this plot the APT data measured on sample set A was rescaled to the lower P-concentrations of sample set E using the P-values shown in Supplementary Fig. S1. The graph shows that the PL intensity is increasingly quenched as function of decreasing mean NC size, although the P-incorporating fraction is almost constant around 50% (indicated by the grey dashed line).

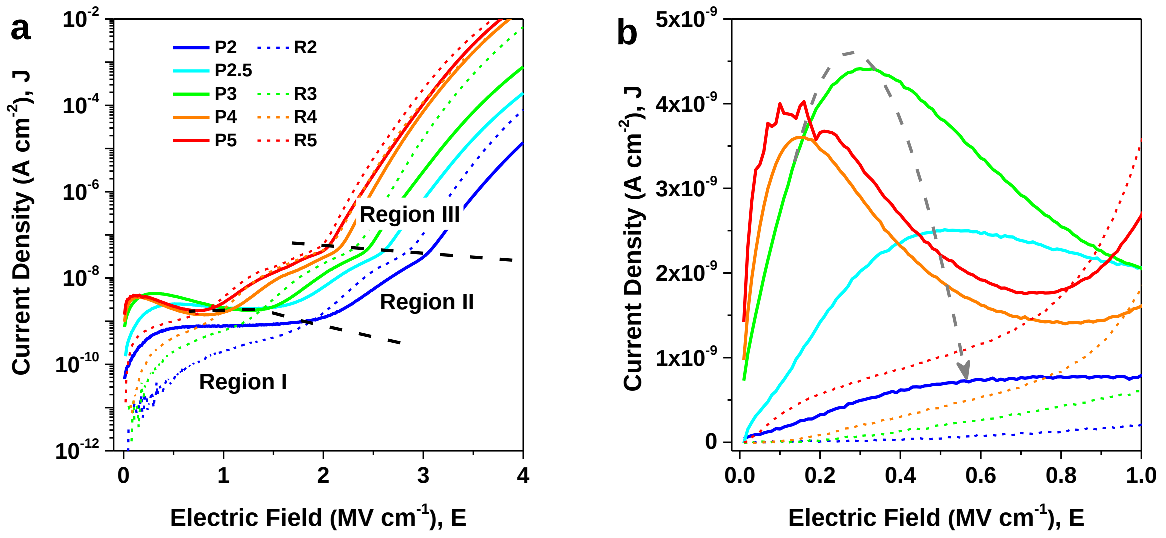

Supplementary Figure S5: (a) J-E plot over the whole bias range (0 – 40 V) of sample set E, i.e., MOS capacitors with thick oxide barriers preventing charge injection from gate or substrate. For all samples we distinguish 3 distinct regions corresponding to different transport regimes, indicated here by black dashed lines:

- Region I: For the undoped reference samples (dashed lines), at low E-fields and in the absence of injected carriers this region is governed by a transient space-charge limited current caused by intrinsic carriers, impurities, or defects. In addition, also some diminutive injection through the oxide via defects cannot be ruled out. For the P-samples we observe in Region I a clear enhancement of J that indicates the presence of additional charge carriers.
- Region II: At medium applied fields we observe for all samples, irrespective of P-doping, a current increase related to dangling bond defect-assisted band-to-band tunnelling from the Si VB to the CB[62]. As described by Gutsch *et al*. this effect is present despite H_2 passivation and deactivation of a majority of dangling bonds at the Si/SiO_2 interface[33], although current densities for unpassivated samples are at least one order of magnitude higher[62].
- Region III: At sufficiently high E-fields the onset of Fowler-Nordheim (FN) tunnelling is observed, i.e., the injection of carriers through the thick oxide barriers.

Since the Si to SiO_2 ratio is decreasing with decreasing average NC size, the onset of all regions is shifted towards higher E-fields. Also, the overall current densities increase with increasing NC size for the same reason. (b) Linear J-E plot showing only the low E-field region of 0 – 1 MV/cm. Clear J-peaks shifted to higher E-fields with decreasing average NC size are visible for all P-samples (indicated by the grey dashed arrow) except for P2. That sample features just an onset of a constant current region starting at ~0.6 MV/cm. Compared to the

well-defined J-peak of sample P3 this is a drastic difference. Therefore, an intermediate size sample (P2.5) was prepared that shows (i) a further shifted J-peak to 0.52 MV/cm and (ii) the transition towards a levelled J-plateau. Hence, the characteristics of P2 are not an artefact but a manifestation of the huge increase in ionization energy and accordingly the high E-field required to delocalize carriers from P-donors. The estimated doping efficiency η_D is similar to the value of sample P3 (~7%). Sample P2.5 was fabricated with 2.5 nm thick SRON:P with C_P = 0.62 at%. The interpolated average number of P-atoms per NC is $[P]_{APT} \approx 1.1$ and the interpolated average NC size is 2.2 nm.

References

13. Borisenko, V. E. & Yudin, S. G. Steady-State Solubility of Substitutional Impurities in Silicon. Phys. Status Solidi A **101**, 123-127 (1987).

17. Nomoto, K. *et al.* Atom probe tomography of size-controlled phosphorus doped silicon nanocrystals. *Phys. Status Solidi RRL* **11**, 1600376 (2017).

33. Jivanescu, M., Hiller, D., Zacharias, M. & Stesmans, A. Size dependence of Pb-type photoluminescence quenching defects at the Si nanocrystal interface. *Europhys. Lett.* **96**, 27003 (2011).

36. Gutsch, S. *et al.* Electronic properties of phosphorus doped silicon nanocrystals embedded in SiO2. *Appl. Phys. Lett.* **106**, 113103 (2015).

42. König, D. Number series of atoms, interatomic bonds and interface bonds defining zinc-blende nanocrystals as function of size, shape and surface orientation: Analytic tools to interpret solid state spectroscopy data. *AIP Adv.* **6**, 085306 (2016).

58. Hellman, O. C., Vandenbroucke, J. A., Rusing, J., Isheim, D. & Seidman, D. N. Analysis of three-dimensional atom-probe data by the proximity histogram. *Microsc. Microanal.* **6**, 437 (2000).

59. Queeney, K. T. *et al.* Infrared spectroscopic analysis of the Si/SiO2 interface structure of thermally oxidized silicon. *J. Appl. Phys.* **87**, 1322-1330 (2000).

60. Zimina, A., Eisebitt, S., Eberhardt, W., Heitmann, J. & Zacharias, M. Electronic structure and chemical environment of silicon nanoclusters embedded in a silicon dioxide matrix. *Appl. Phys. Lett.* **88**, 163103 (2006).

61. Hernández, S. *et al.* Determining the crystalline degree of silicon nanoclusters/SiO2 multilayers by Raman scattering. *J. Appl. Phys.* **115**, 203504 (2014).

62. Gutsch, S. *et al.* Charge transport in Si nanocrystal/SiO2 superlattices. *J. Appl. Phys.* **113**, 133703 (2013).
